# The glioma neuron symbiosis hypothesis—cellular and molecular mechanistic considerations

**DOI:** 10.3389/fnins.2026.1815478

**Published:** 2026-05-08

**Authors:** Avital Schurr

**Affiliations:** Department of Anesthesiology and Perioperative Medicine, University of Louisville, School of Medicine, Louisville, KY, United States

**Keywords:** glioma, glucose, GLUT, lactate, lactylation, LDH, MCT, neuron

## Abstract

A recent hypothesis suggests that glioma cells and neurons engage in a symbiotic relationships, where neurons tend to use lactate, produced in abundance by the cancer cells, instead of glucose. Consequently, the glucose conserved by neurons becomes accessible to glioma cells, which have a high demand for it. The present monograph further develops this hypothesis, weighing specific cellular and molecular processes in both cell types that allow for these symbiotic relationships. The potential roles in the postulated symbiosis of the glycolytic pathway, the mitochondrial tricarboxylic acid cycle, and its coupled oxidative phosphorylation, glucose and lactate transporters, the excitatory neurotransmitter glutamate, lactate signaling via its receptor, and lactylation, are all considered here. The aim is to provide a wider foundation with greater detail for a better understanding of the proposed symbiosis that could offer several possible experimental avenues to verify its validity.

## 1 Introduction

The glioma neuron symbiosis (GNS) hypothesis recently proposed (Schurr, 2025) is based on two scientific discoveries made decades apart. The first described the phenomenon of aerobic glycolysis in carcinoma cells (Warburg, 1925, 1956), a process that produced lactate at a much higher rate than normal cells. The second showed for the first time that lactate is oxidatively utilized as a sole energy substrate to support neuronal function (Schurr, 1988). Warburg effect, the high rate of glycolytic activity, is a hallmark of all cancerous tumors, including cancers of the brain. Among the latter, gliomas are the most common. As our knowledge of cellular energy producing processes advances, the ability of cancerous cells to take advantage of the surrounding normal cells and their microenvironment has received great attention. This is especially true where glioma cells are concerned (Cuddapah et al., 2014; Gillespie and Monje, 2018; Crivii et al., 2022; Venkataramani et al., 2022). The GNS hypothesis postulates that both cell types, glioma cells and neurons, benefit from their interactions. Accordingly, neurons preferentially use the abundance of lactate produced by the glioma cells for their own mitochondrial ATP production. In return, the spared neuronal glucose should be sufficed for the neuronal pentose phosphate pathway (PPP) (Diaz-Garcia and Yellen, 2018; TeSlaa et al., 2023) and enough glucose leftover for the glycolytic activity of the glucose craving glioma cells. For both neurons and glioma cells to maintain their symbiotic relationships, various cellular processes and molecular entities must participate. Accordingly, glioma cells secrete lactate that is taken up by neurons. These shuttles of lactate occur via specific transporters known as monocarboxylate transporters (MCTs). Similarly, neurons either secrete their spared glucose out or slow down transporting it, making it available for glioma cells. These processes utilize specific glucose transporters (GLUT1, GLUT3). The metabolic pathways that allow the consumption of lactate and glucose, by neurons and glioma cells, respectively, should operate optimally, i.e., the mitochondrial oxidative phosphorylation (OXPHOS) in the former and glycolysis in the latter. In addition, beyond being an oxidative energy substrate, lactate is also known as a signaling molecule, operating via the hydroxycarboxylic acid receptor 1 (HCAR1). The metabolism of lactate requires its oxidation to pyruvate by mitochondrial lactate dehydrogenase (mLDH), an enzymatic reaction that takes place in neurons, although known to also occur in glioma cells. This monograph explores the potential role each of these cellular processes and molecular components play in the postulated symbiosis between glioma cells and neurons and offers experimental possibilities to prove or disprove such a role. Seeking treatment or cure is the main driving force behind cancer research which is not the focus of this article. Its specific thrust is to highlight the possible molecular mechanisms involved in a hypothetical symbiotic relationship between glioma cells and neurons. One could speculate further on the possible role of neurons in the pathogenesis of glioma due to these postulated relationships, but first the existence of symbiosis needs experimental verification. The experimental avenues offered here aim at investigating the contribution of glioma-produced lactate environment to the hypothesized symbiosis. Any of the proposed experimental avenues could interfere with the postulated symbiosis and therefore provide an insight into the role of neurons in glioma proliferation and pathogenesis.

## 2 Cellular elements and processes that could play a role in GNS

### 2.1 Glucose transporters and transport

The membranal transporters responsible for the transportation of both glucose and lactate are of the facilitated diffusion type, allowing the passage of these molecules through the cell (plasma) and organelle membranes along the concentration gradient. Due to the high demand of glucose by cancerous cells for the glycolytic production of adenosine triphosphate (ATP), the transportation of glucose via its transporters becomes a rate limiting factor. A recent publication offered an in-depth examination of glucose transporters found in normal and cancer cells (Chamarthy and Mekala, 2023). The role of two of these transporters, GLUT1 and GLUT3, in controlling glucose movements in and out of glioma cells and neurons will be the focus here, although studies also show a role for GLUT4, especially in nerve terminals during synaptic activity (Kobayashi et al., 1996; Vannucci et al., 1998; Ashrafi et al., 2017). GLUT1 activity is much higher in cancer cells than in normal cells (Salter et al., 1982; Flier et al., 1987; Birnbaum et al., 1987; Sistonen et al., 1989). Glioblastoma cells show elevated levels of both GLUT1 and GLUT3 expression. The neuronal transporter is GLUT3 that has a lower *K*_m_ and a higher *K*_cat_, i.e., stronger affinity for and faster rate of glucose transport than GLUT1 (Simpson et al., 2007). GLUT1 is the transporter in glial cells, including astrocytes, which is responsible for glucose uptake from the extracellular space (Swansom and Graham, 1994). This distinction is important since there are similarities between glioma cells and glia (Moreno-Sánchez et al., 2007). Where glioma cells are concerned, studies have shown that the GLUT1 and GLUT3 proteins are upregulated (Parson et al., 2008; Marin-Valencia et al., 2012; Clark et al., 2016). Could transcription changes in GLUT3 activity occur in neurons entangled in symbiotic relationships with glioma cells? Could the abundance of glioma-produced lactate bring about a decrease in neuronal GLUT3 synthesis and activity due to the decrease in neuronal glucose consumption? Such changes would be responsible for the postulated neuronal glucose sparing and its availability for consumption by glioma cells (Schurr, 2025).

**Figure 1:**
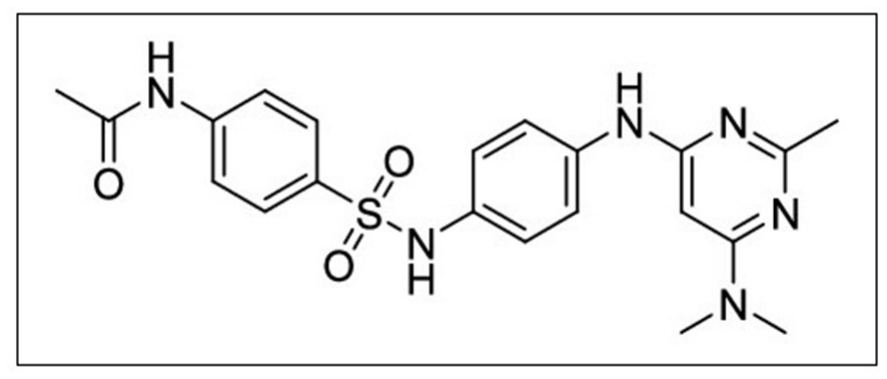
The chemical structure of the specific GLUT3 inhibitor G3iA [20].

To verify changes in GLUT3 activity in neurons due to their symbiosis with glioma cells and the high concentration lactate environment, one must postulate that the activity of GLUT3 decreases under such conditions. A differentiated PC12 cell line culture serves as an effective testing system, with cell cultures maintained in two types of media: a normal glucose medium (control) and a lactate-enriched medium (experimental). Following a fixed incubation period, the GLUT3 activity of the neurons cultured in the lactate-enriched medium should decrease in comparison to neurons in the glucose-fed, control culture, a decrease quantified by measuring the entry of [^13^C] Glucose into neurons in each of the two cultures. Another approach is to assess how a selective GLUT3 inhibitor influences neuron survival in each culture. It is necessary to evaluate each culture in conditions where both glucose and lactate are available. Neurons cultured with lactate should remain healthy, whereas those grown on glucose alone are likely to decrease significantly in number. A promising inhibitor candidate for that purpose is G3iA (Figure 1), a GLUT3 specific inhibitor with IC_50_ = 7.01 ± 1.02 μM (Iancu et al., 2022). Many experimental anticancer therapies aim to block GLUT, with a range of compounds developed and tested for this purpose (Wang et al., 2025). To address the symbiotic relationship between glioma cells and neurons, it may be beneficial to use multiple inhibitors, each targeting a specific transporter: GLUT1 in glioma cells and GLUT3 in neurons. The goal is to disrupt this symbiosis as part of anticancer therapy.

The postulated neuronal glucose sparing does not assume that glioma-produced lactate makes glucose consumption by neurons obsolete. Normally, glucose is the main glycolytic substrate that produces the mitochondrial substrate of the tricarboxylic acids (TCA) cycle, including during neuronal activity (Li et al., 2023), and as mentioned above, also for the PPP. However, neurons and glioma cells shared microenvironment cannot be considered normal conditions, where the abundance of lactate makes it the preferred oxidative substrate for neurons (Hu and Wilson, 1997; Schurr et al., 1999; Qu et al., 2000; Mangia et al., 2003; Smith et al., 2003; Kasischke et al., 2004; Schurr, 2025). Moreover, the ability of glioma cells to interact with and activate glutamatergic neurons via the secretion of glutamate (Gillespie and Monje, 2018) should increase neuronal activity and oxidative consumption of lactate (see section 4).

### 2.2 Lactate transport and transporters

An extensive review of the structure, function, and regulation of the monocarboxylate transporters (MCTs) is available (Halestrap and Price, 1999). Subsequent research revealed that these transporters are part of the *SLC16* gene family (Halestrap and Meredith, 2004). The increased glycolytic activity of cancerous cells drives a rise in lactate production that must extrude out of these cells through MCT1 and MCT4. Evidence shows that the heightened lactate levels increase the expression of *MCT1* and *MCT4* genes (Miranda-Gonçalve et al., 2017; Longhitano et al., 2022). According to the GNS hypothesis (Schurr, 2025) lactate, shown to be the preferred neuronal substrate of the mitochondrial OXPHOS (Smith et al., 2003; Bouzier-Sore et al., 2003, 2006; Schurr, 2006; Wyss et al., 2011; Karagiannis et al., 2021; Dembitskaya et al., 2022), becomes the main energy substrate for neurons that interact with glioma cells. The main lactate transporter in neurons is MCT2 (Pierre et al., 2002; Pierre and Pellerin, 2005; Medel et al., 2022) and its expression could increase in neurons interacting with glioma cells when exposed to a constant supply of lactate in a similar mode to the effect of lactate on MCT1 and MCT4 in glioma cells. Additionally, research indicates that lactate supports angiogenesis (Lee et al., 2026), which could enhance glucose delivery to glioma cells and increase oxygen availability for neurons that metabolize lactate. Moreover, researchers proposed an oncologic remodeling hypothesis suggesting that lactate plays a role in promoting biosynthesis and influencing epigenetic changes in cancer (Aydin, 2025). That very idea could pertain to neurons in the immediate, lactate-rich microenvironment of glioma cells, i.e., an increased expression of *MCT2* and its protein to accommodate for the ample supply of lactate for oxidative ATP production and slowdown in neuronal glycolysis. Lactate is not just an energy substrate and a metabolite; it is also a signaling molecule that has its own receptor (Cai et al., 2008; Lauritzen et al., 2014; Wu et al., 2021). Hydroxycarboxylic acid 1 (HCAR1) receptors have been identified in the brain, including within regions like the hippocampus and the neocortex (Lauritzen et al., 2014). The increased expression of MCT1 and MCT4 by lactate signaling via HCAR1, believed to promote glioma cells proliferation, could easily be duplicated by neurons to increase their MCT2 expression. Moreover, lactate induces the expression of HCAR1 itself (Longhitano et al., 2022), an induction that may occur in neurons, too.

**Figure 2:**
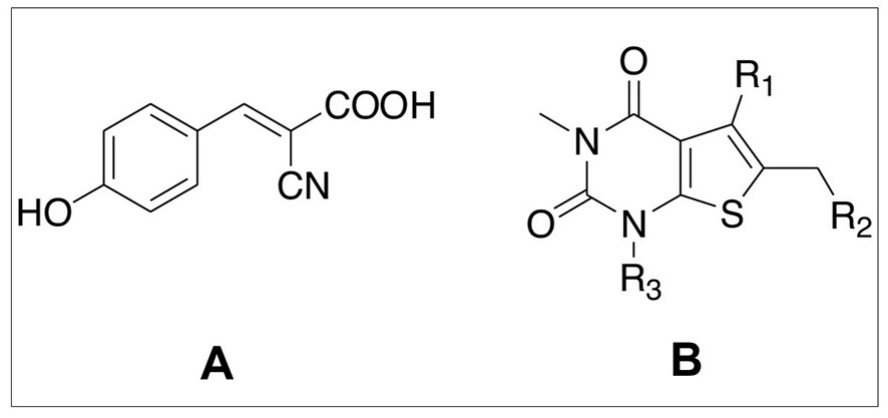
The chemical structures of **(A)** the MCT inhibitor α-cyano-4-hydroxy-cinnamic acid (4CIN) and **(B)** AZD3965. The R groups in the latter are: R_1_ = (S)-4-hydroxy-4-methyl-isoxazolidine-2-carbonyl, R_2_ = (3-trifluoromethyl-5-methyl-1H-pyrazol-4-yl) methyl, R_3_ = isopropyl.

The changes that lactate abundance induces in glioma cells and neurons postulated to interact with them, where MTCs are concerned, could be evaluated using the very cell lines suggested for testing induced changes in GLUTs. One should expect to document a significant increase in MCT2 activity in differentiated PC12 neurons cultured in lactate-enriched medium compared to control neurons cultured in glucose-supplemented medium. This alteration can be assessed using [^13^C] lactate. Moreover, these lactate-maintained neurons should be more sensitive to the presence of a specific MCT2 blocker when compared to control neurons incubated in a medium containing both glucose and lactate. Unfortunately, there are unknown MCT2 inhibitors that have no inhibiting effect on either MCT1 or MCT4, the main two MCTs also found in glioma cells and astrocytes. However, MCT2 appears to be more sensitive to certain inhibitors than the other two MCTs (Morland et al., 2015). One of these inhibitors is α-cyano-4-hydroxy-cinnamic acid (4CIN) (Figure 2A), with a K_i_ = 24 μM for lactate transport by MCT2, while the inhibition constants for MCT1 and MCT4 are K_i_ = 166 μM and K_i_ = 994 μM, respectively. When considering the proposed symbiotic relationship between glioma cells and neurons, effective anticancer MCT inhibitor candidates should target both MCT1 and MCT2. This approach would block lactate from leaving glioma cells and prevent its entry into neurons. The inhibitor, AZD3965, seems to be, for now, the most effective in this regard (Figure 2B).

### 2.3 Lactate dehydrogenases LDHA and LDHB

There are five different isomers of the LDH enzyme. These isomers differ from one another by the composition of their four subunits, a composition that determines the direction of the reaction they catalyze, pyruvate to lactate or *vice versa*. Two genes, *LDHA* and *LDHB*, found in most tissues and organs including the brain, code for LDH proteins. LDHA converts pyruvate to lactate, while LDHB converts lactate to pyruvate (Valvona et al., 2016). The enzyme is a tetramer that could be composed of four subunits, all being the product of either the *LDHA* gene or the *LDHB* gene (homotetramers) or any combination between the two for a total of five different possible combinations. LDH isomers composed of three or four LDHA subunits show higher affinity to pyruvate and its conversion to lactate, while isomers that contain three or four LDHB subunits show higher affinity to lactate and its conversion to pyruvate. LDHA, the final enzyme of the glycolytic pathway, resides in the cytosol, like the rest of the glycolytic complex. In contrast, LDHB resides in the mitochondrion, where it oxidizes lactate to pyruvate (Schurr and Payne, 2007; Atlante et al., 2007; Hashimoto et al., 2008). Pyruvate undergoes conversion into acetyl CoA, which then serves as the starting material for the tricarboxylic acid (TCA) cycle. Lactate increases the production of LDHA in cancerous cells by upregulation of the *LDHA* expression (Passarella et al., 2014; Altinoz and Ozpinar, 2022; Huang et al., 2025), promoting higher glycolytic rate and proliferation of glioma cells. Lactate, when available abundantly for neurons, should, according to the GNS hypothesis, increase the production of LDHB in neuronal mitochondria via the upregulation of the *LDHB* gene, as the monocarboxylate becomes the main energy substrate.

**Figure 3:**
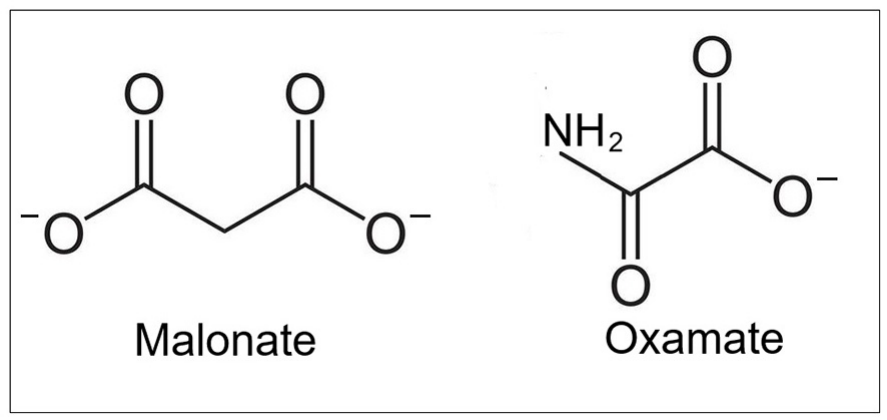
The chemical structure of malonate and oxamate, a relatively specific LDHB inhibitors (Schurr and Payne, 2007).

The differentiated PC12 cell neurons grown in a medium rich in lactate would, again, be an adequate system to confirm changes in LDH activity. These neurons should exhibit upregulation of the *LDHB* neuronal gene to increase LDHB production in neuronal mitochondria. Nevertheless, LDH is an enzyme for which only a limited number of potent inhibitors are known. Moreover, no known inhibitors distinguish between LDHA and LDHB. One study found a measurable difference in specificity or potency between two LDH inhibitors (Schurr and Payne, 2007). These were oxamate, which was expected to show a higher potency toward LDHA, but were found to be a better LDHB inhibitor, and malonate, which exhibited higher potency toward LDHB (Figure 3). While malonate is known as a succinate dehydrogenase inhibitor that could disrupt mitochondrial respiration and the TCA cycle, it was not the case in that *in vitro* study (Schurr and Payne, 2007). Using the acute hippocampal slice preparation, malonate suppressed neuronal function when lactate was the oxidative mitochondrial substrate, by inhibiting its conversion to pyruvate by LDHB. In contrast, when pyruvate was used as the oxidative substrate, neuronal function was undisturbed and no effect on LHDA activity was detected. In the same study, oxamate, a known LDH inhibitor, had similar effect to malonate on lactate-supported neuronal function, while being harmless when pyruvate was the substrate. Therefore, oxamate could be used in the proposed experiments if the malonate inhibition of succinate dehydrogenase is of concern. Consequently, the sensitivity of differentiated PC12 cell line of differentiated neurons cultured in lactate enriched medium to LDHB inhibition by either malonate or oxamate should be higher than the sensitivity of control culture maintained in glucose medium.

### 2.4 Glutamate

The potential effect of glutamate on both glioma cells and neurons, especially excitatory ones, deserves additional consideration. Originally, the GNS hypothesis postulated a role for glutamate secreted by glioma cells as an excitation mechanism of neurons that increases their lactate consumption to support their elevated energy needs (Ye and Sontheime, 1999; Buckingham et al., 2011; Campbell et al., 2012; Venkataramani et al., 2022; Schurr, 2025). The hypothesis also weighed in on the similarities between astrocytes and glioma cells (Pan and Monje, 2022). As a result, it is crucial to examine how glutamate, released by excitatory neurons, could affect glioma cells, particularly since this neurotransmitter is known to rapidly increase glucose uptake in astrocytes via GLUT1 (Loaiza et al., 2003). However, the many roles glutamate plays in neurons, glia (astrocytes) and glioma cells, and its potential effects on the various receptors and transporters (Pellerin and Magistretti, 1994; de Groot and Sontheimer, 2011; Souza et al., 2013) would make assessment of the role of this excitatory neurotransmitter in the postulated symbiotic relationships between glioma cells and neurons, at least at this stage of the GNS hypothesis development, too complex, if not confusing. Nevertheless, the experiments suggested for testing the effect of lactate on both GLUTs and MCTs activity and expression in differentiated PC12 neurons may also be performed in the presence of glutamate in the culture medium.

### 2.5 Lactylation

Recently, researchers have identified lactylation as a type of post-translational protein modification to be a significant process in the development and proliferation of cancer (Zhang et al., 2019; Chen et al., 2021; Wan et al., 2022; Dai et al., 2024). Lactylation of proteins, histones and non-histones alike, occurs both enzymatically and by simple lactate binding. Considering the lactate-enriched microenvironment of glioma cells and their adjacent neurons, lactylation of neuronal proteins could play its own role in promoting the symbiotic relationships between the two cell types. Most lactylation research centers on its functions and potential links to cancer, due to the Warburg effect. Where lactylation of proteins in non-cancerous cells, the data available relies on abnormal conditions such as hypoxia or sepsis. It is plausible that neurons interaction with glioma cells, especially at the initial stages of such interaction, involves neuronal post-translational protein lactylation that could affect the expression and activity of transporters and enzymes that participate in oxidative lactate metabolism. While further studies are necessary to determine if lactylation contributes to neuronal activity in the symbiotic interactions between glioma cells and neurons, researchers can explore this process by cultivating differentiated PC12 cell line neurons in a medium enriched with lactate. A method is available to detect lysine L-lactylation (Zhang et al., 2025), the primary form of lactylation observed on histones, within neurons exposed to high levels of lactate.

## 3 Discussion

The GNS hypothesis (Schurr, 2025) is postulating a symbiotic relationships between glioma cells and neurons, especially at the preliminary stages of malignancy. The hypothesis proposes that the need by cancer cells to rid themselves of the copious amounts of lactate they produced (Warburg effect), is drawn them to neurons that are known to preferentially use lactate as their mitochondrial substrate for ATP production. By utilizing lactate, neurons spare their own glucose, making it available for the hexose-hungry glioma cells. The hypothesis suggested various experimental approaches to test it, yet it lacked an in-depth exploration of the cellular and molecular mechanisms that might play a role in these symbiotic relationships. This essay outlines cellular and molecular mechanisms that may contribute to the proposed GNS. In addition, it should provide a better understanding of the hypothesis and routes to exploit potential treatments of glioma and other malignancies. The two major molecular components that must be involved in exchanging lactate for glucose between glioma cells and neurons are the membranal transporters through which these two energy substrates cross in and out of cells and organelles. Therefore, the activity of both GLUT1 and GLUT3 should be involved as major molecular components of the symbiotic relationships between glioma cells and neurons, respectively. Similarly, the expression and activity of the corresponding monocarboxylate transporters, MCT1 and MCT4 in glioma cells, and MCT2 in neurons, should increase in both cell types. As glioma cells and neurons exchange glucose and lactate, their metabolic pathways need to be able to process higher substrate concentrations efficiently. Glioma cells rely on glycolysis for glucose processing, while neurons use the TCA cycle and OXPHOS to metabolize lactate. The rate-limiting enzymatic step affects both LDHA in cytosolic glycolysis within glioma cells and LDHB in the mitochondrial conversion of lactate to pyruvate in neurons. In both cases, pyruvate is subsequently converted to acetyl CoA, which enters the TCA cycle. The excitatory neurotransmitter glutamate could have its own effects on both cell types, excite neurons to consume more lactate, while enhancing glucose uptake in glioma cells. To determine whether the phenomenon of protein lactylation does occur in neurons residing within the microenvironment of glioma cells, and the possible role it may play in the postulated symbiosis between the two cell types require further research. Every cellular and molecular component believed to contribute to the development and progression of gliomas should be involved not only within the glioma cells themselves but also in the neurons present in their microenvironment. Therefore, experimental suggestions are made to test for the possible presence of these cellular and molecular changes in neuronal population exposed to lactate-enriched enviroment that mimics the microenvironment conditions neurons are under according to the GNS hypothesis.

## 4 Conclusions

The present essay details various major transformations in cellular, molecular, and metabolic mechanisms that take place in glioma cells, transformations that could also occur to one extent or another in neurons with which the glioma cells interact. Such changes in both cell types, if they occur, should reveal the foundation on which their symbiotic relationships rely. A better understanding of changes both in transport and metabolism of the two energy substrates, glucose in glioma cells and lactate in neurons, and the proposed GNS could lead to potential anticancer treatments.

## Data Availability

The original contributions presented in the study are included in the article/supplementary material, further inquiries can be directed to the corresponding author.
